# Mutations in the SPAST gene causing hereditary spastic paraplegia are related to global topological alterations in brain functional networks

**DOI:** 10.1007/s10072-019-3725-y

**Published:** 2019-02-08

**Authors:** Rosaria Rucco, Marianna Liparoti, Francesca Jacini, Fabio Baselice, Antonella Antenora, Giuseppe De Michele, Chiara Criscuolo, Antonio Vettoliere, Laura Mandolesi, Giuseppe Sorrentino, Pierpaolo Sorrentino

**Affiliations:** 1 Department of Science and Technology, University of Naples Parthenope, Naples, Italy; 2 Institute of Applied Sciences and Intelligent Systems, CNR, Pozzuoli, Italy; 3 Department of Motor Sciences and Wellness, University of Naples Parthenope, Via Medina 38, 80133 Naples, Italy; 4 Hermitage-Capodimonte Hospital, Naples, Italy; 5 Department of Engineering, University of Naples Parthenope, Naples, Italy; 6 Department of Neurosciences, Reproductive, and Odontostomatological Sciences, University of Naples Federico II, Policlinico Hospital, Building 17, Via S. Pansini 5, 80131 Naples, Italy; 7 Department of Humanistic Studies, University of Naples Federico II, Naples, Italy

**Keywords:** Hereditary spastic paraplegia, Motoneuron disease, Magnetoencephalography, Magnetic source imaging, Brain network, Neural synchronization

## Abstract

**Aim:**

Our aim was to describe the rearrangements of the brain activity related to genetic mutations in the SPAST gene.

**Methods:**

Ten SPG4 patients and ten controls underwent a 5 min resting state magnetoencephalography recording and neurological examination. A beamformer algorithm reconstructed the activity of 90 brain areas. The phase lag index was used to estimate synchrony between brain areas. The minimum spanning tree was used to estimate topological metrics such as the leaf fraction (a measure of network integration) and the degree divergence (a measure of the resilience of the network against pathological events). The betweenness centrality (a measure to estimate the centrality of the brain areas) was used to estimate the centrality of each brain area.

**Results:**

Our results showed topological rearrangements in the beta band. Specifically, the degree divergence was lower in patients as compared to controls and this parameter related to clinical disability. No differences appeared in leaf fraction nor in betweenness centrality.

**Conclusion:**

Mutations in the SPAST gene are related to a reorganization of the brain topology.

## Introduction

Hereditary spastic paraplegias (HSPs) are a group of inherited neurological diseases with a highly complex and heterogeneous clinical profile. Based on the clinical phenotype, HSPs have been classified into pure and complex (complicated) forms. The former is characterized by pyramidal signs affecting predominantly the lower limbs [1]. In the latter, the core symptoms are associated with a wide spectrum of additional neurological and extraneurological signs [2], including mental retardation, peripheral neuropathy, cerebellar ataxia, epilepsy, optic atrophy, retinitis pigmentosa, deafness, and cataracts. Such wide clinical variability partially reflects the underlying genetic backgrounds. To date, 76 loci and 58 corresponding genes [spastic paraplegia genes (SPGs)] have been linked to HSPs [3–6].

Spastic paraplegia type 4 (SPG4) is caused by mutations in the SPAST gene (located on 2p22.3), which encodes for an enzyme called spastin [7, 8]. SPG4 is the most common autosomal-dominant form of HSP, accounting for approximately 40% of familial [9] and 10% of sporadic [10] cases. SPG4 is classically considered a “pure” form of HSP and the clinical phenotype is dominated by a slowly progressive paraparesis with an insidious onset [9, 11] but without diminished life expectancy. However, recent clinical and neuroimaging studies have shown that SPG4 patients also manifest cognitive impairment [12, 13], cerebellar ataxia [14], thin corpus callosum [12], or lower motor neuron dysfunction [15]. Interestingly, a magnetic resonance multimodal study addressing structural connectivity in 11 patients affected by SPG4 and 23 controls identified microstructural damage in the corticospinal tracts, in the anterior cingulate cortex, and in the splenium of the corpus callosum [16]. Very recently, Liao et al. have addressed resting state functional connectivity in SPG4 using fMRI [17], showing widespread increased connectivity in patients as compared to controls. However, there is no conclusive evidence on the actual spread of the neurodegenerative process in SPG4.

In the present work, we set out to study the functional alterations linked to mutations in the SPAST gene. More specifically, accordingly to ours [18] and other authors’ [19] previous studies, we hypothesized that the pathological process induces a global reorganization of the brain functional networks. To test our hypothesis, we applied the phase lag index (PLI), which quantifies synchronization between time series, followed by the minimum spanning tree (MST) algorithm, which allows the computation of statistically comparable metrics [20], to magnetoencephalography (MEG) data obtained from a cohort of SPG4 patients and healthy controls. Finally, we estimated network characteristics of the patients and healthy controls and related them to clinical disability. By selecting patients with a specific form of spastic paraplegia, this study is also meant as a proof of concept to link a genetic feature (i.e., mutations in SPG4) to frequency-specific brain network properties.

## Materials and methods

### Participants and clinical assessment

Ten patients (eight males and two females) from seven families with diagnosis of HSP according to Harding criteria [3] and with molecular confirmation of SPG4 were enrolled [21]. All the patients showed only sign of involvement of the pyramidal system. Ten age-, gender-, and education-matched controls were also analyzed. Exclusion criteria were a family and/or personal history of neurologic and psychiatric disease. The mean age of our cohort was 53.6 years ± 11.6 (40–74 years), and the mean age at onset of symptoms was 36.2 ± 10.3 (18–50 years). All treatments that might interfere with brain connectivity, especially antispasmodic, had been suspended from 3 weeks before acquisition. The degree of disability was rated using the Spastic Paraplegia Rating Scale (SPRS). More characteristics of the patients are reported in Table 1. This study complied with the Declaration of Helsinki and was approved by the local ethics committee “Comitato Etico Campania Centro” (Prot.n.93C.E./Reg. n.14-17OSS). Written informed consent has been given by all participants.

**Table 1:** Characteristics of the patients

#	Gender	Age at examination	Age at onset	Mutation
1	M	54	43	del 2–17
2	M	63	46	c.1310delTATAA
3	M	46	30	c.1728+1G>A
4	M	40	38	c.1728+1G>A
5	F	65	50	del 1–17
6	M	44	35	c.373G>T
7	M	74	25	del 8–16
8	M	48	17	del 8–16
9	M	41	33	IVS11+3delAAGT
10	F	61	45	IVS11+3delAAGT

### Acquisition

All participants underwent a magnetoencephalographic examination in a 163-magnetometer MEG system, developed by the National Research Council, at the Institute of Applied Sciences and Intelligent Systems “E. Caianiello”, Pozzuoli, Naples. Before the acquisition, four positions coils were attached on the subject’s head and were digitalized using Fastrak (Polhemus®). The coils were activated, and localized, at the beginning of each segment of registration. The subject was seated on a comfortable armchair placed in the shielded room. Electrocardiographic and electrooculographic signals were co-recorded to aid artifact removal. Brain activity was recorded during resting state for two distinct segments of 2.5 min with eyes closed. MEG data, after an anti-aliasing filter, were acquired with a sampling frequency of 1024 Hz. The signal was then filtered using a fourth order Butterworth IIR band-pass filter in the 0.5–48 Hz band.

### Preprocessing

Firstly, principal component analysis (PCA) was used to reduce environmental noise. We adopted the PCA filtering implementation available within the fieldtrip toolbox [22]. Subsequently, noisy channels were removed manually through visual inspection by an experienced rater. Finally, for each subject, supervised independent component analysis (ICA) was used to remove physiological, cardiac (generally one component), and blinking (if present) artifacts from the MEG signals. The first ten epochs of 8 s for each subject that did not contain artifacts (either system-related or physiological) or strong environmental noise were selected. The length of 8 s is a trade-off between the need to have enough cleaned epochs, to avoid drowsiness [23], and to obtain a reliable estimate of the connectivity measure [24].

### Source reconstruction

All the processing related to the beamforming procedure has been done using the FieldTrip toolbox [22]. Based on an MRI template, the volume conduction model proposed by Nolte [25] was considered and the linearly constrained minimum variance (LCMV) beamformer [26] was implemented to reconstruct time series related to the centroids of 116 regions-of-interest (ROIs), derived from the automated anatomical labeling (AAL) atlas [27, 28]. We considered only the first 90 ROIs, excluding those corresponding to the cerebellum given the low reliability of the reconstructed signal in those areas. Source space-time series were re-sampled at 512 Hz.

### Connectivity estimation

The connectivity estimation was performed using BrainWave software [CJS, version 09.152.1.23, available from http://home.kpn.nl/stam7883/brainwave.html]. The epochs were band-pass filtered into five frequency bands (delta (0.5–4 Hz), theta (4.0–8.0 Hz), alpha (8.0–13.0 Hz), beta (13.0–30.0 Hz), and gamma (30.0–48.0 Hz)) and PLI was computed [29], retrieving a 90 × 90 adjacency matrix for each epoch of each subject in each frequency band. The PLI is based on the distribution of the differences of the instantaneous phases (derived from the Hilbert transformation of the times series) for two time series. This measure is insensitive to signal leakage [30] (at the cost of discarding true zero–lag interactions). PLI values range between 0 and 1, where 1 indicates perfect synchronization and 0 indicates non-synchronous activity.

### Graph theoretical analysis

The weighted adjacency matrices were used to reconstruct a functional network, with the 90 regions of the AAL atlas are represented as nodes and the 1/PLI values as weighted edges. For each frequency band, a MST was constructed using Kruskal’s algorithm [31]. The MST is a loopless graph with N nodes and M = N-1 links. The MST was used to obtain topologic measures that are unaffected by the degree distribution, matrix density, or arbitrary thresholds [20, 32]. After this step, we computed both global and nodal parameters which provide insight respectively on the global features of the network and on the centrality of each of the 90 ROIs. Of the global parameters, we calculated leaf fraction (L) and degree divergence (K). L is defined as the fraction of nodes with degree of 1 [33], providing an indication of the integration of the network. A higher L implies that the network tends toward a star-like topology, where the nodes are on average closer to each other as compared to a more line-like topology. The K is a measure of the broadness of the degree distribution, related both to the resilience against pathological events and to the synchronizability of the networks [20]. Of the nodal parameters, we calculated the betweenness centrality (BC) which represents the number of shortest paths passing through a given node, divided by the total number of shortest paths of the network [33]. BC is a measure to estimate the centrality of the brain areas.

**Fig. 1:**
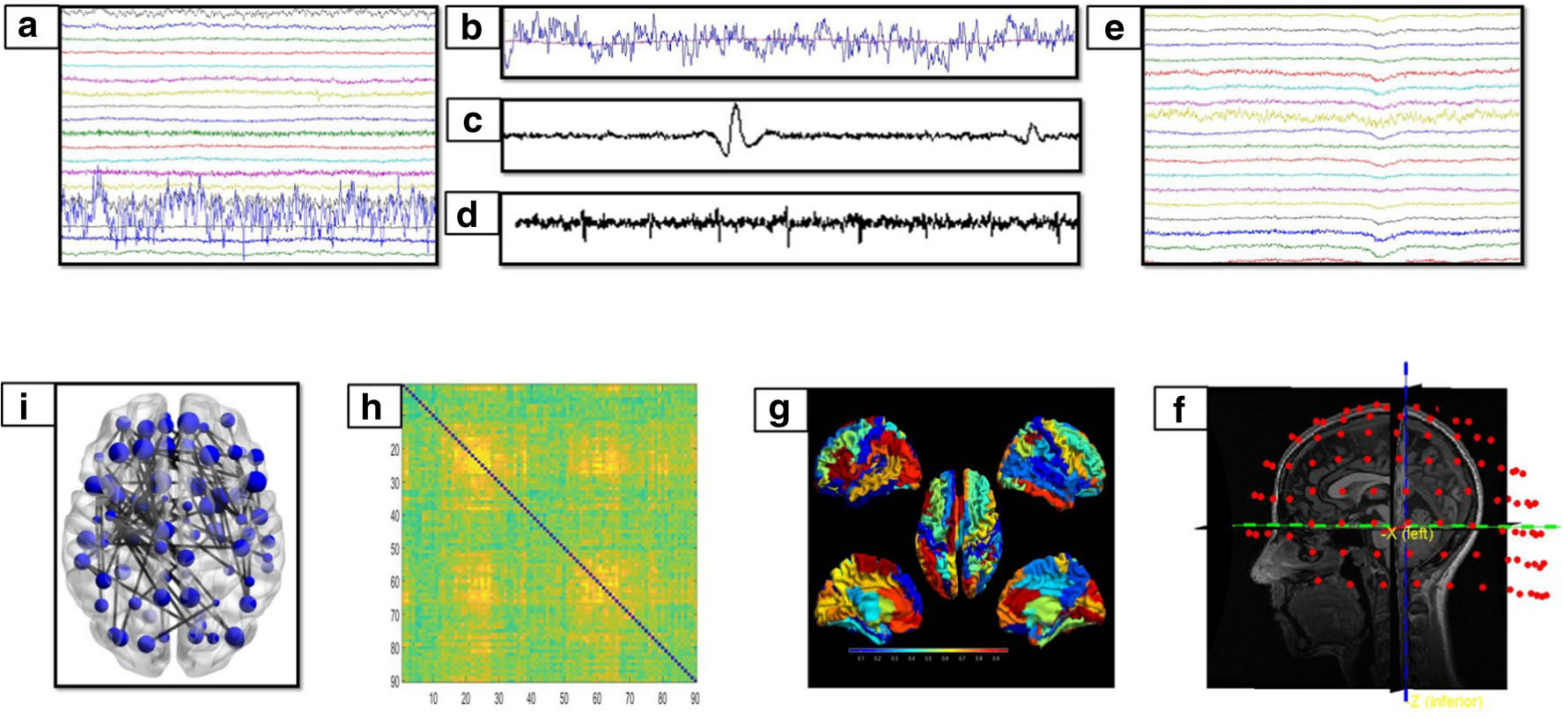
Data analysis pipeline. **a** Raw MEG signals recorded by 154 sensors (a subset displayed here). **b**–**d** Respectively noisy channel, cardiac artifact, blinking artifact, removed during pre-processing phase. **e** MEG signals after artifact removal and noise cleaning. **f** Co-registration between MEG signals and MRI template. **g** Source reconstruction (beamforming). **h** Functional connectivity matrix estimated for each frequency band (delta, theta, alpha, beta, gamma) using the PLI. Rows and columns are the regions of interest, while the entries are the estimated values of the PLI. **i** Brain topology representation based on the MST

All these metrics were calculated for each epoch and subsequently, averaged across epochs for each subject separately. An overview of the whole pipeline is provided in Fig. 1.

### Statistical analysis

Permutation analyses were performed in Matlab (MathWorks, version R2013a). The global network metrics (L and k) were averaged per subject (i.e., averaged over epochs) in each frequency band. Statistical significance was assessed by permuting the subject’s labels 10,000 times [34]. A significance level of 0.05 was used, after false discovery rate (FDR) correction across multiple metrics and frequency bands. A similar procedure was used when analyzing the BC results, but the *p* values were corrected also across areas with FDR [35]. In case they were significantly different, the parameters were related to clinical disability (tested using the SPRS scale) by Pearson coefficient correlation using IBM SPSS.

## Results

**Fig. 2:**
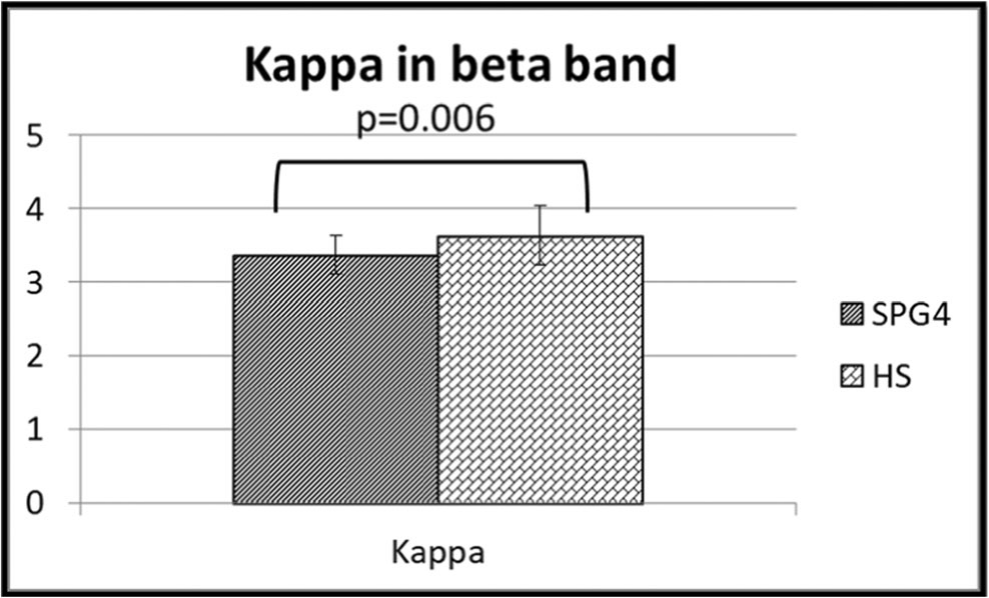
Changes in global parameters. Differences of the degree divergence (K) in the beta band between SPG4 patients (SPG4) and controls (HS)

**Fig. 3:**
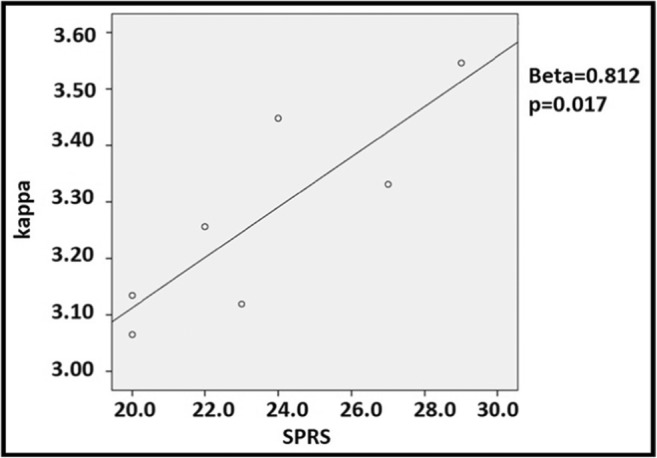
Pearson coefficient correlation between degree divergence (K) and SPRS rate. Pearson coefficient correlation between the values at Spastic Paraplegia Rating Scale and the degree divergence (K) in eight SPG4 patients. Two subjects have the same values for degree divergence and for SPRS, so in the figure, they are superimposed. Two outliers have been excluded from the analysis

Our results showed no difference in BC, between patients, and healthy controls. The broadness of the degree divergence appeared to be lower in the networks of patients as compared to controls, specifically in the beta band (*p* = 0.0003, after multiple comparisons correction across metrics and frequency bands *p* = 0.006) (Fig. 2). Furthermore, in SPG4 the K was directly related to the SPRS (Beta = 0.812, *p* = 0.017). This analysis was performed excluding two outliers (Fig. 3). No difference was evident when comparing the leaf fraction between groups.

## Discussion

This is the first study to address frequency-specific brain functional network alterations in SPG4 using MEG. We set out to test if SPG4, a form of spastic paraplegia which is traditionally considered “pure,” displays topological alterations in functional brain networks. We found that in the beta band, the degree divergence appears to be lower in patients as compared to controls. Degree divergence is proportional to the mean degree squared. Hence, in a minimum spanning tree, a lower degree divergence implies that nodes have lower degrees more often and, hence, the network is less connected and less prone to synchronization [20]. Such global network rearrangements might be related to the widespread phenotypical alterations going beyond motor impairment. Indeed, recent evidence shows that in SPG4, neurodegeneration might be present beyond the pyramidal tracts, as in the cerebellum, neocortex, corpus callosum, and optic nerve [36]. Interestingly, both demyelination and axonal damage can result in changes of both local and long-range connections, resulting in functional alterations in the alpha and beta bands [37]. The interhemispheric coupling in the beta band correlates directly with motor recovery in patients after stroke [38]. Despite a modified global topology, no difference in BC was found in specific brain areas. In this line, previous studies showed that the presence of a risk factor for Alzheimer’s disease, such as high IGF-1 serum levels, does not relate to alterations in specific areas, but rather to global topological rearrangements in the beta and theta bands [39].

Finally, we showed that the degree divergence directly relates to the clinical disability. This is a somewhat surprising result. A speculative interpretation of this observation could be that, in case of pathology, hubs failure is partly compensated by the rerouting of traffic to more peripheral nodes. The presence of more nodes with lower degrees might be capturing such compensation [18]. The extent of such compensation relates to a better clinical condition (as measured by lower scores in the SPRS scale). In other words, the more the disease causes disconnection, the more nodes with low degree become frequent in the network in order to compensate for the loss of nodes with high degree. Similar compensatory mechanisms have been frequently proposed. For example, very recently, Liao et al. in SPG4 patients have interpreted the increased neuronal activity in the precentral gyrus as a “compensatory moderator of diseases symptoms” [17].

However, given the small size of our sample, such conclusion needs to be confirmed. Nevertheless, this reflects the rarity of these conditions, and it is in line with previous neuroimaging studies in these patients [17, 40, 41].

One more issue to take into account is that the PLI does not remove fully the effects of secondary leakage [42].

In conclusion, our results show that genetic modification in SPG4 might carry a specific signature in terms of frequency-specific rearrangements of the functional brain networks.
